# Behavioral Modeling of Dynamic Nonlinear Distortions in 5G Wireless Transmitters Using Cascaded Augmented Real-Valued Neural Networks

**DOI:** 10.3390/s26123832

**Published:** 2026-06-16

**Authors:** Sharafa Bankole, Reem Alnajjar, Majid Ahmed, Souheil Bensmida, Oualid Hammi

**Affiliations:** 1 Department of Electrical Engineering, College of Engineering, American University of Sharjah, Sharjah P.O. Box 26666, United Arab Emirates; 2 Institute of Sensors, Signals and Systems, School of Engineering and Physical Sciences, Heriot-Watt University, Edinburgh EH14 4AS, UK

**Keywords:** 5G, behavioral model, distortions, neural networks, nonlinear distortions, real valued time delay neural network (RVTDNN), wireless infrastructure

## Abstract

**Highlights:**

**What are the main findings?**

- Dynamic nonlinear distortions in 5G wireless transmitters can be accurately modeled using a cascade made of two specialized neural networks.
- Using cascaded neural networks for the behavioral modeling of dynamic distortions in 5G wireless infrastructure can lead to reduced overall complexity without loss of accuracy.

**What are the implications of the main findings?**

- The proposed model and the reported results show that instead of building a massive complex neural network to handle the entire dynamic nonlinear behavior, it is more effective to use dedicated models with fewer coefficients.
- By significantly reducing the number of parameters while maintaining the model’s accuracy, the proposed architecture can contribute to the adoption of neural networks in field deployed systems with resource constrained hardware.

**Abstract:**

Neural networks are increasingly adopted for performance enhancement in wireless communication infrastructure for 5G and 6G applications. This paper proposes a modular two-box neural network-based system for the behavioral modeling of dynamic nonlinear distortions observed in wireless transmitters. The proposed model, labeled cascaded augmented real-valued artificial neural networks (CAR-VANN), uses a first neural network with an augmented but memoryless input vector feature to model memoryless nonlinear behavior. This model is designed for low-complexity and coarse estimation of the nonlinear distortions. The second neural network, which aims to fine-tune the model output and boost its accuracy, is a conventional augmented real-valued time-delay neural network (ARVTDNN). Experimental validation shows that the CAR-VANN model can achieve the same performance as the ARVTDNN with a significant reduction in the number of parameters (between 35% and 52%). Accordingly, this model can be considered a viable alternative for the computationally efficient modeling of dynamic nonlinear distortions in 5G systems, reducing the computational complexity associated with neural networks-based models without compromising their performance.

## 1. Introduction

The cornerstone of modern intelligent systems is seamless, high-speed, and high-capacity wireless connectivity, where communication infrastructure serves as the backbone, enabling a wide array of applications. Fifth Generation (5G) communication systems and beyond, including the envisioned 6G, are the key enabling technologies for this always-visible and connected ecosystem, providing ultra-low latency, massive device density, and ultra-reliable connectivity. To achieve the desired level of performance, wireless communication systems are required to operate linearly to preserve the integrity of transmitted signals and avoid interference, while also maintaining high power efficiency to reduce their carbon footprint. A critical and pivotal subsystem in communication infrastructure is the radio frequency (RF) power amplifier (PA). Due to their nonlinear behavior, PAs introduce both in-band distortions and spectral regrowth, which degrade signal quality and spectral efficiency. These effects become more pronounced as modern communication systems continue to evolve toward wider bandwidths, higher-order modulation schemes, and increasingly stringent spectral emission requirements. In this context, it is essential to develop accurate behavioral models that can predict the level of distortions caused by a power amplifier to assess its impact on the overall communication system. Furthermore, this can be used to develop distortion compensation mechanisms such as digital predistorters [1].

Conventional implementations of power amplifiers’ behavioral models are typically based on analytical functions derived from Volterra series expansions or simplified polynomial structures [2,3,4,5,6,7,8]. While these approaches provide satisfactory performance for mildly nonlinear PAs, their complexity rapidly increases when strong memory effects are present and wideband signals are considered. To address the complexity limitations of single-box behavioral models and predistortion functions, cascaded and multi-box structures have been widely investigated [1,9,10,11,12,13,14]. These architectures exploit the fact that a PA’s behavior can be decomposed into memoryless and dynamic nonlinear behaviors. By distributing the modeling task across multiple stages, multi-box-based models, such as Hammerstein and Wiener models [9,10], as well as twin-nonlinear two-box (TNTB) models [11], achieve improved trade-offs between modeling accuracy and computational complexity.

More recently, artificial neural networks (ANNs) have gained significant attention for PA behavioral modeling and predistortion due to their strong generalizability, and adaptability. Dense neural networks (DNNs) [15,16,17,18,19], convolutional neural networks (CNNs) [20,21,22], recurrent neural networks (RNNs) [23,24,25], and attention-based networks [26] have all been reported to outperform classical analytical models in terms of modeling accuracy. However, this performance advantage is often accompanied by a substantial increase in computational complexity, parameter count, and training cost. These challenges are further exacerbated with wideband signals, where memory effects are stronger and more difficult to model.

To reduce the computational cost associated with ANN models, two-box ANN-based architectures have been introduced. These approaches extend the classical two-box modeling philosophy to neural networks by separating memoryless and memory dependent nonlinearities across two sub-models. An example of this is presented in [27], which employed a memoryless LUT to compensate for static distortions and a bidirectional LSTM network to model memory effects. Although such architecture achieved strong modeling performance, it remains computationally expensive due to the use of recurrent structures and bidirectional processing in the neural network sub-model.

Motivated by the need for low-complexity ANN behavioral models, this work proposes a cascaded augmented real-valued neural network architecture for PA behavioral modeling. The proposed model follows a two-box formulation in which the first neural network is memoryless and designed to provide a coarse estimation of the PA static nonlinear behavior, and the second neural network is an augmented real-valued time delay neural network responsible for modeling the residual dynamic nonlinear behavior. The proposed CAR-VANN model aims at reducing the complexity of previously proposed two-box neural network models by using a feedforward neural network rather than a recurrent neural network. Compared to analytically defined two-box behavioral models (such as twin-nonlinear two-box models and Hammerstein and Wiener models), the use of neural networks-based two-box models is expected to enhance the modeling capabilities owing to the inherent advantages of neural networks. The remainder of this paper is organized as follows. Section 2 introduces the proposed architecture and describes its model structure and identification procedure. Section 3 presents experimental setup and performance evaluation. Finally, Section 4 concludes the paper.

## 2. Cascaded Augmented Real-Valued Neural Networks

### 2.1. Model Structure

**Figure 1:**
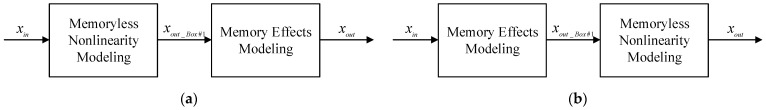
Cascaded two-box arrangement of behavioral models: (**a**) Hammerstein and forward TNTB-like configuration; (**b**) Wiener and reverse TNTB-like configuration.

In this work, a two-box neural network structure is proposed for the behavioral modeling of the distortions exhibited within modern communication systems due to the nonlinearity of power amplifiers. The proposed model is inspired from previously reported two-box structures for analytically defined models [11]. In these models, the behavior of the power amplifier is modeled by cascading a memoryless function with a second function that models memory effects. There are two possible arrangements for cascaded two-box models used for modeling nonlinear distortions in presence of memory effects. These two arrangements are depicted in Figure 1. The first configuration, comparable to the Hammerstein and forward TNTB models, employs a first function that aims at modeling the static (i.e., memoryless) distortions of the device under test (DUT), followed by a second function dedicated to the modeling of the memory effects. This configuration is shown in Figure 1a. Conversely, the second configuration, illustrated in Figure 1b, uses a function designed to model the memory effects followed by a second function used to model the memoryless nonlinearity. This arrangement is similar to the Wiener and the reverse TNTB models. Both configurations have been proposed for modeling as well as compensating the nonlinear distortions and memory effects caused by power amplifiers in wireless communication systems and were found to lead to comparable performance. However, the use of the Hammerstein and forward TNTB configuration is more suitable for behavioral modeling applications due to its inherent ease of identification when compared to the other configuration. Furthermore, the Wiener-like and reverse TNTB configuration is more convenient for digital predistortion applications due to its suitability for sequential linearization [14] and its ability to be used along with reduced observation bandwidth [13].

**Figure 2:**
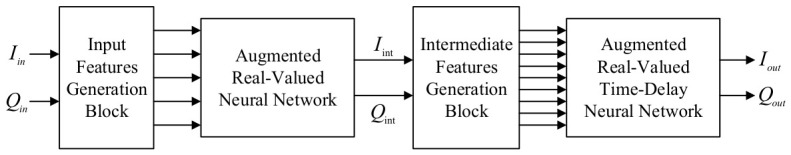
Simplified block-diagram of the proposed cascaded augmented real-valued neural networks model.

The proposed model, which is devised for the behavioral modeling of dynamic nonlinear distortions, uses the forward TNTB model configuration. The simplified block-diagram of the proposed model is depicted in Figure 2. It is made of an augmented real-valued neural network (ARVNN) followed by an augmented real-valued time-delay neural network. Hence, the proposed model is referred to as the cascaded augmented real-valued artificial neural networks (CAR-VANN). The first neural network is memoryless whereas the second incorporates memory effects modeling through the use of the time-delay structure. The model includes two features shaping blocks. The input features shaping block is used to generate the input features vector that will be fed to the ARVNN by including terms that depend on the input signal’s magnitude. The intermediate features shaping block has a similar function; however, it is set to also generate delayed terms of the signal at its input to model the memory effects. By having two neural networks with controllable complexity dedicated to the memoryless nonlinearity and the memory effects modeling, the proposed model is expected to reduce overall complexity without compromising modeling accuracy, as is typical with two-box models. This is because static and dynamic distortions exhibit fundamentally different behaviors. Memoryless distortions are typically strongly nonlinear and depend only on the instantaneous sample, whereas memory effects are generally less nonlinear and depend on past samples.

In Figure 2, the signals $I_{in}$ and $Q_{in}$ represent the in-phase and quadrature components of the model’s input signal $x_{in}$, respectively. Similarly, the signals $I_{out}$ and $Q_{out}$ represent the in-phase and quadrature components of the model’s output signal $x_{out}$. The outputs of the first neural network are $I_{int}$ and $Q_{int}$, namely, the in-phase and quadrature components of the intermediate signal $x_{int}$.

**Figure 3:**
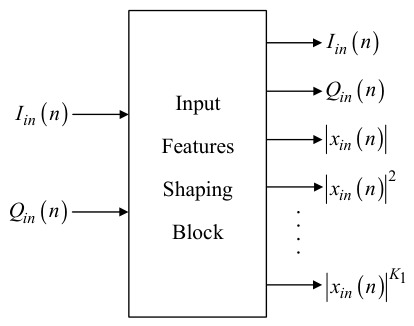
Input features shaping block.

The input features shaping block will generate the input features vector $X_{1}$ for the first neural network. $X_{1}$ is given by

(1) $$X_{1}\left(n\right)=\left[\begin{array}{l} I_{in}\left(n\right)\\ Q_{in}\left(n\right)\\ \left|x_{in}\left(n\right)\right|\\ {\left|x_{in}\left(n\right)\right|}^{2}\\ \;\;\;\;\;\vdots \\ {\left|x_{in}\left(n\right)\right|}^{K_{1}} \end{array}\right],$$

where $K_{1}$ represents the highest magnitude order to be used in the input vector of the first neural network. Figure 3 depicts the inputs and outputs of the input features shaping block.

**Figure 4:**
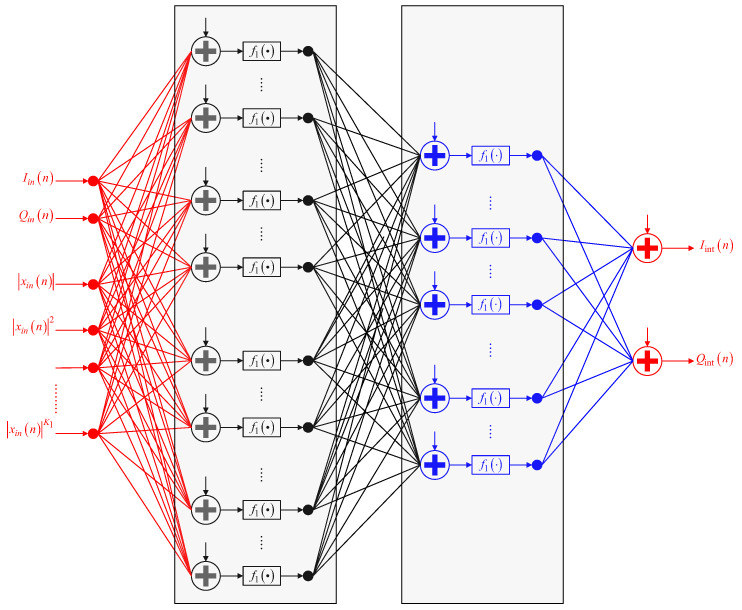
Block diagram of the augmented real-valued densely connected neural network.

The outputs of the input features shaping block are then applied at the input of the first neural network. A detailed block diagram of the augmented real-valued densely connected network used in this work is presented in Figure 4. For ease of representation, this figure shows the case of a network with two hidden layers.

The in-phase and quadrature components $I_{int}$ and $Q_{int}$ of the complex baseband signal, $x_{int}$, at the output of the first neural network are fed into the intermediate features shaping block to generate the input vector $\left(X_{2}\right)$ of the second neural network. $X_{2}$ can be defined as

(2) $$X_{2}\left(n\right)=\left[\begin{array}{l} I_{\mathrm{int}}\left(n,M_{0}\right)\\ Q_{\mathrm{int}}\left(n,M_{0}\right)\\ \left|x_{\mathrm{int}}\left(n,M_{1}\right)\right|\\ {\left|x_{\mathrm{int}}\left(n,M_{2}\right)\right|}^{2}\\ \;\;\;\;\;\vdots \\ {\left|x_{\mathrm{int}}\left(n,M_{K_{2}}\right)\right|}^{K_{2}} \end{array}\right],$$

where $K_{2}$ represents the highest magnitude order to be used in the input vector of the second neural network, and

(3) $$I_{\mathrm{int}}\left(n,M_{0}\right)={\left[I_{\mathrm{int}}\left(n\right)\;\;\;I_{\mathrm{int}}\left(n-1\right)\;\;\;\cdots \;\;\;I_{\mathrm{int}}\left(n-M_{0}\right)\right]}^{T},$$

(4) $$Q_{int}\left(n,M_{0}\right)={\left[Q_{\mathrm{int}}\left(n\right)\;\;\;Q_{\mathrm{int}}\left(n-1\right)\;\;\;\cdots \;\;\;Q_{\mathrm{int}}\left(n-M_{0}\right)\right]}^{T},$$

and for $j=1,2,\cdots ,K_{2}$,

(5) $${\left|X_{int}\left(n,M_{j}\right)\right|}^{j}={\left[{\left|x_{\mathrm{int}}\left(n\right)\right|}^{j}\;\;\;{\left|x_{\mathrm{int}}\left(n-1\right)\right|}^{j}\;\;\;\cdots \;\;\;{\left|x_{\mathrm{int}}\left(n-M_{j}\right)\right|}^{j}\right]}^{T},$$

**Figure 5:**
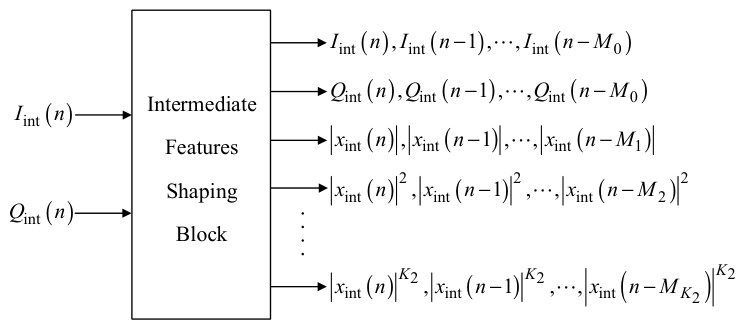
Intermediate features shaping block.

Figure 5 illustrates the inputs and outputs of the intermediate features shaping block. The outputs of the intermediate features shaping block constitute the inputs of the second neural network. The structure of the second network is similar to that depicted in Figure 3 for the first neural network. The only difference is related to the input features and their number.

As can be seen in Equation (2), the memory depth associated with each of the input features of the second neural network can be customized and set to different values. This is an important aspect, since it is known that memory effects are mainly linear; hence, the impact of higher-order nonlinear terms on the modeling of memory effects will be decaying. This is achieved in the proposed model by ensuring that $M_{i+1}\leq M_{i}$ for $i=0,1,\cdots ,K_{2}-1$.

Reducing the memory depth for higher-order terms will translate into a smaller number of input features and therefore a more compact neural network without affecting the modeling performance.

As the proposed model is made up of two cascaded neural networks, its complexity depends on the number of layers and neurons used for each network. To elaborate, the total number of input features to the first neural network is

(6) $$N_{0,ARVNN}=K_{1}+2,$$

where $K_{1}$ is the nonlinearity order used in the first ARVNN of the proposed model.

Consequently, the overall complexity of the ARVNN will be given by

(7) $$C_{ARVNN}=\sum_{i=0}^{L_{ARVNN}}\left(N_{i,ARVNN}+1\right)N_{i+1,ARVNN},$$

where $N_{i,ARVNN}$ represents the number of neurons in the *i*th layer of the ARVNN, and $L_{ARVNN}$ in the number of hidden layers in the ARVNN. The input and output layers correspond to i=0 and $i=L_{ARVNN}+1$, respectively.

Similarly, the total number of input features to the augmented real-valued time delay neural network is

(8) $$N_{0,ARVTDNN}=2\left(M_{0}+1\right)+\sum_{i=1}^{K_{2}}\left(M_{i}+1\right),$$

Hence, the number of parameters in the ARVTDNN is

(9) $$C_{ARVTDNN}=\sum_{i=0}^{L_{ARVTDNN}}\left(N_{i,ARVTDNN}+1\right)N_{i+1,ARVTDNN},$$

where $N_{i,ARVTDNN}$ represents the number of neurons in the *i*th layer of the ARVTDNN, and $L_{ARVTDNN}$ in the number of hidden layers in the ARVTDNN. The input and output layers correspond to i=0 and $i=L_{ARVTDNN}+1$, respectively.

Finally, the total number of parameters in the CAR-VANN model is

(10) $$C=\sum_{i=0}^{L_{ARVNN}}\left(N_{i,ARVNN}+1\right)N_{i+1,ARVNN}+\sum_{i=0}^{L_{ARVTDNN}}\left(N_{i,ARVTDNN}+1\right)N_{i+1,ARVTDNN},$$

Table 1 summarizes the variables used for each of the two neural networks and their respective definitions.

### 2.2. Model Identification

**Figure 6:**
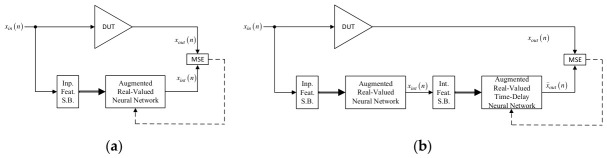
Identification of the proposed CAR-VANN model: (**a**) identification of the first NN; (**b**) identification of the second NN.

The identification process of the proposed CAR-VANN model is made of the two steps depicted in Figure 6. This is conceptually similar to the process commonly used for identifying analytically defined two-box models. First, the memoryless network is trained to approximate the behavior of the power amplifier. In this step, the focus is on achieving a balance between complexity and performance. The model complexity is defined in terms of its number of parameters (weights and biases), and the model performance is quantified in terms of the normalized mean-squared error (NMSE). For a given activation function, the number of layers and the number of neurons per layer are swept to determine a suitable size of the augmented real-valued neural network that leads to a trade-off between complexity and performance.

Once the first model is trained, its parameters (including the values of the weights and biases) are frozen, the intermediate signal is computed and used to generate the input features of the second neural network. Then, a second training process, illustrated in Figure 6b, occurs during which only the weights and biases of the second neural network are trained. By the end of this step, the two-box model is fully identified, and its performance can be assessed.

### 2.3. Training Protocol

The PA measurement dataset is partitioned into training, validation, and test subsets in a 60%/20%/20% ratio. For the 40 MHz test signal, the total dataset comprises 153,593 samples, yielding 92,156 training samples, 30,719 validation samples, and 30,719 test samples. All models are trained using the Adam optimizer without a fixed random seed. The training dataset is continuously compared with the validation dataset throughout training to monitor performance and prevent overfitting. The complete training parameters are summarized in Table 2, showcasing the swept parameters for each neural network, which include the number of hidden layers, the number of neurons in each hidden layer, and the activation functions utilized. The cost function utilized is the mean squared error (MSE), which is given by

(11) $$MSE=\frac{1}{N}\sum_{n=1}^{N}{\left|y\left(n\right)-\overset{⌢}{y}\left(n\right)\right|}^{2},$$

where y represents the target complex output waveform being predicted by the model (actual measured complex baseband output waveform), and $\overset{⌢}{y}$ is the predicted waveform corresponding to y. N is the number of samples in the these waveforms.

The detailed training methodology of the proposed model is summarized in Algorithm 1. The sequential identification procedure is outlined such that the first neural network, that is the ARVNN, is trained first with a fixed set of hyper parameters, followed by the second neural network, the ARVTDNN, which is trained using the intermediate output resulting from the first box. Both neural networks are trained to the maximum number of epochs, whilst retaining the model weights that minimize the validation loss. The settings used for the training of all neural networks reported in this work are summarized in Table 2.
**Algorithm 1.** Two-Stage Identification of the CAR-VANN Model**Input:** $x_{in}$$\mathrm{and}\;x_{out}$: measured PA input and output signals                              $\;I_{in},\;Q_{in},\;I_{out},\;Q_{out}$ for *N* samples.1:Chosen Box 1 (ARVNN) hyperparameters: 
      $K_{1}$: nonlinearity order;     $\;L_{ARVNN}$: number of hidden layers;     $\;N_{i,ARVNN}$: number of neurons per layer;     $\;f_{1}$: activation function.2:Chosen Box 2 (ARVTDNN) hyperparameters: 
      $K_{2}$: nonlinearity order;     $\;{\left\{M_{i}\right\}}_{i=0}^{K_{2}}$: memory depths;     $\;L_{ARVTDNN}$: number of hidden layers;     $\;N_{i,ARVTDNN}$: number of neurons per layer;     $\;f_{2}$: activation function.3:Training settings
      η: learning rate;     $\;E_{\max}$: training epochs.**Output:** $\theta_{1}^{*}$ optimal weights and biased of the ARVNN;              $\;\theta_{2}^{*}$: optimal weights and biased of the ARVTDNN.4:$\mathrm{Partition}\;x_{in}$ $\mathrm{and}\;x_{out}$ into 60% training, 20% validation, and 20% testing sets.5:$X_{1}\leftarrow$ $\mathrm{input}\;\mathrm{features}\;\mathrm{shaping}\;\mathrm{of}\;x_{in}$$\mathrm{with}\;\mathrm{order}\;K_{1}$ (Equation (1)).6:$\theta_{1}^{*}\leftarrow$ $\mathrm{Train}\;(\mathrm{ARVNN},\;X_{1},$ $x_{out}$, chosen box 1 parameters, η, $E_{\max}$).7:$\mathrm{Freeze}\;\theta_{1}^{*};$ $\mathrm{compute}\;\mathrm{intermediate}\;\mathrm{signal}\;x_{\mathrm{int}}=ARVNN\left(X_{1};\theta_{1}^{*}\right)$ $\mathrm{components}\;I_{\mathrm{int}}$ $\mathrm{and}\;Q_{\mathrm{int}}.$8:$X_{2}\leftarrow$ $\mathrm{intermediate}\;\mathrm{features}\;\mathrm{shaping}\;\mathrm{of}\;x_{int}$ $\mathrm{with}\;\mathrm{order}\;K_{2}$ $\mathrm{and}\;\mathrm{memory}\;\mathrm{depths}\;{\left\{M_{i}\right\}}_{i=0}^{K_{2}}$ (Equation (2)).9:$\theta_{2}^{*}\leftarrow$$\mathrm{Train}\;(\mathrm{ARVTDNN},\;X_{2},$ $x_{out}$, chosen box 2 parameters, η, $E_{\max}$).10:$\mathrm{Report}\;NMSE_{test}$ of the full cascade on the test set.11:$\mathrm{Return}\;\theta_{1}^{*}$ $\mathrm{and}\;\theta_{2}^{*}.$

## 3. Performance Assessment and Validation

### 3.1. Experimental Setup and Benchmark Model

**Figure 7:**
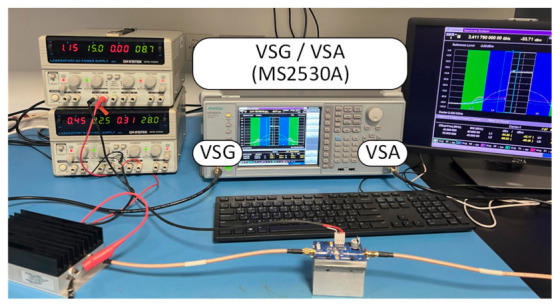
Photograph of the experimental setup.

The proposed model’s efficacy was verified using the experimental setup shown in Figure 7. This setup is made of the MS2530A vector signal generator/vector signal analyzer from Anritsu, Kanagawa, Japan. The vector signal generator module of this instrument is used to generate the RF signal from the digital baseband waveform, and the vector signal analyzer module acquires the RF signal at the output of the device under test and provides access to the digital baseband output waveform. The baseband signals used for the model identification had a total duration of 1 ms each. The digital baseband input and output waveforms are then used to devise the behavioral models of the DUT. The device under test is a Gallium Nitride (GaN)-based PA with a small signal gain of 13 dB. The DUT was tested using a 5G compliant test signal generated using MATLAB’s (2024) 5G toolbox. This signal has a 40 MHz bandwidth with a peak to average power ratio (PAPR) of 10 dB. The signal was sampled at 153.6 Msps. The tests were performed at a center frequency of 2593 MHz, which corresponds to the center frequency of the n41 band of the 5G NR standard. During the tests, the PA was operated at an output power back-off that is equal to the signal’s PAPR to ensure that the DUT is characterized over its entire power range.

The performance of the models was evaluated using the normalized mean squared error (NMSE) which is given by

(12) $$NMSE=10\log_{10}\left(\frac{\sum_{n=1}^{N}{\left|y\left(n\right)-\overset{⌢}{y}\left(n\right)\right|}^{2}}{\sum_{n=1}^{N}{\left|y\left(n\right)\right|}^{2}}\right),$$

where y, $\overset{⌢}{y}$ and N represent the same variables as in Equation (11).

The ARVTDNN benchmark model was evaluated by adjusting the number of parameters and the activation function. The number of layers was varied from 1 to 4, and for each layer, the number of neurons was swept from 5 to 40 in steps of 5. Three activation functions were considered. These are the rectified linear unit (ReLU), the Tanh, and the Sigmoid functions, as defined in Equations (13)–(15), respectively.

(13) $$f_{\mathrm{ReLU}}\left(x\right)=\left\{\begin{array}{l} x\;\mathrm{if}\;x>0\\ 0\;\mathrm{if}\;x\leq 0 \end{array}\right.,$$

(14) $$f_{\tanh}\left(x\right)=\frac{e^{x}-e^{-x}}{e^{x}+e^{-x}},$$

(15) $$f_{sig}\left(x\right)=\frac{1}{1+e^{-x}},$$

**Figure 8:**
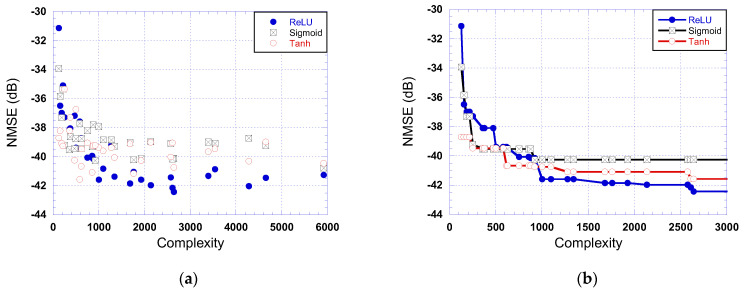
Performance assessment of the single-box ARVTDNN: (**a**) NMSE; (**b**) decaying NMSE.

The input vector of the ARVTDNN was generated according to Equation (2) with a nonlinearity order $K_{2}=3$ and memory depths of $M_{0}=5$, $M_{1}=4$, $M_{2}=3$, and $M_{3}=0$. The model performance was assessed for 96 different settings as summarized in Table 3. The performance of the ARVTDNN benchmark model as a function of its complexity is reported in Figure 8. Here, the complexity represents the total number of trainable parameters in the neural network including weights and biases as given by Equation (9). Figure 8a shows the NMSE results for all considered cases, while Figure 8b depicts the decaying NMSE profiles obtained by keeping the neural network sizes that result in an improvement in the NMSE as the complexity increases. Figure 8b demonstrates that the ReLU activation function leads to the best performance of approximately −42.4 dB with a total of 2642 parameters while the best NMSE obtained with Tanh activation function is limited to −41.6 dB achieved with a minimum of 2612 parameters. Furthermore, at low-complexity (up to 1000 parameters), the Tanh activation function outperforms the ReLU and Sigmoid activation functions. However, for higher-complexity models, the ReLU activation function results in an additional 1 dB to 2 dB performance gain in the NMSE compared to the Sigmoid and Tanh functions, respectively. For the single-box ARVTDNN, the complexity needed for each activation function selection to achieve an NMSE better than −40 dB is summarized in Table 4.

**Figure 9:**
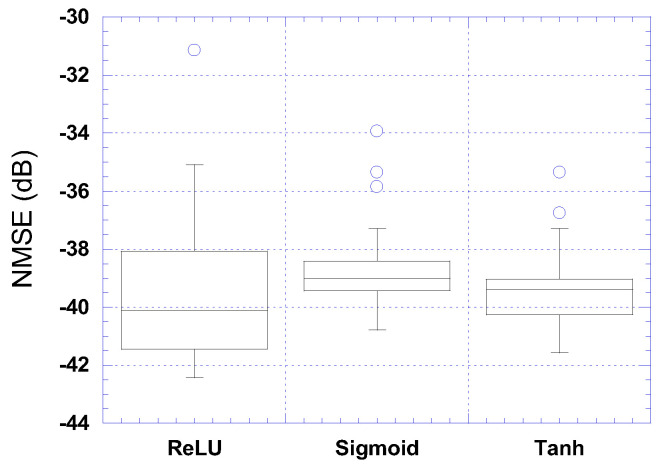
Box-plot of the single-box ARVTDNN NMSE for the three activation functions.

Figure 9 shows the box-plot for the NMSE of the ARVTDNN model for the three activation functions. These results confirm the superiority of the ReLU activation function. This figure also shows that the spread of the NMSE performance (excluding outliers) is much smaller for the Sigmoid activation function. In fact, when the Sigmoid activation function is used, increasing the model complexity results in minor enhancement in the NMSE when compared to the case of the ReLU activation function.

### 3.2. CAR-VANN Model Validation

**Figure 10:**
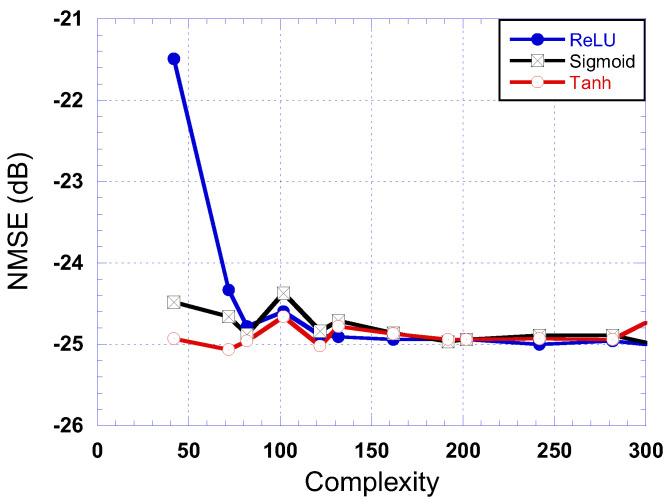
NMSE performance of the CAR-VANN first box for various activation functions.

As described in the previous section, the first step for deriving the CAR-VANN model is the training of the memoryless augmented real-valued NN and the selection of its hyperparameters, including the number of layers, the number of neurons per layer, and the activation function. For this first box, the input features were generated using $K_{1}=3$. The neural network’s hyperparameters were swept in a manner identical to that reported in Table 3. The NMSE results as a function of model complexity are summarized in Figure 10. This figure shows that for all activation functions, the NMSE converges to its best performance for a relatively low complexity (less than 100 parameters). Most importantly, this shows that the memoryless model performance is capped to approximately −25 dB, which clearly demonstrates the strong memory effects exhibited by the DUT. The number of layers and the number of neurons per layer selected for the first box of the CAR-VANN model are reported in Table 5 along with the corresponding performance and complexity.

Each of the three models reported in Table 5 was then used to build the CAR-VANN model by adding the second neural network as described in steps (7) to (10) of Algorithm 1. Here also, a sweep on the parameters of the ARVTDNN parameters was performed in accordance with the settings summarized in Table 3. Hence, for each of the three ARVNN reported in Table 5, 96 ARVTDNN were trained. For consistency and fair comparison with the benchmark model, the input vector of the second box of the CAR-VANN model was built using the same features and settings as the benchmark model; that is, $K_{2}=3$, $M_{0}=5$, $M_{1}=4$, $M_{2}=3$, and $M_{3}=0$.

**Figure 11:**
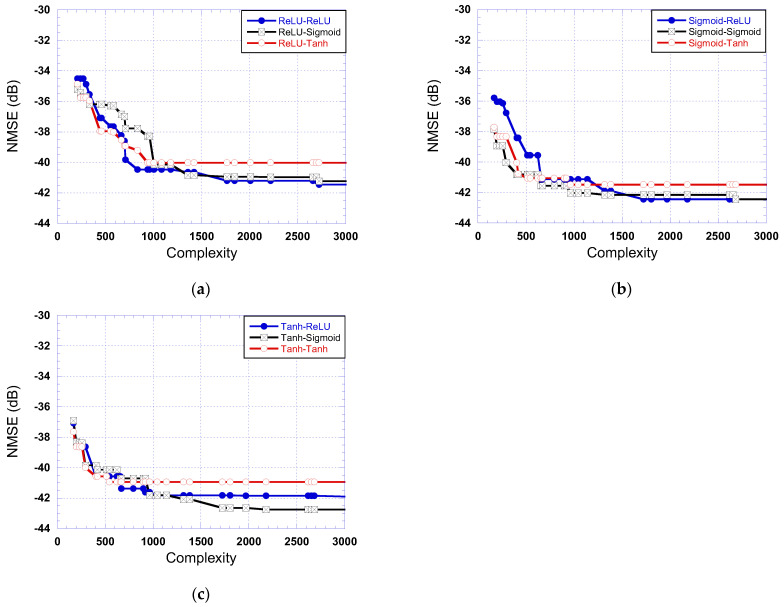
NMSE performance of the CAR-VANN model. (**a**) First-box using ReLU activation function. (**b**) First-box using Sigmoid activation function. (**c**) First-box using Tanh activation function.

The overall performance of the CAR-VANN model is reported in Figure 11. These results show that using the Tanh activation function in the second box of the proposed CAR-VANN model consistently leads to limited performance when compared to other combinations of activation functions. Furthermore, the use of the Sigmoid activation function in the second box appears to consistently lead to satisfactory performance. To compare the proposed model with the ARVTDNN benchmark, Table 6 reports the minimum complexity needed to achieve an NMSE better than −40 dB. This NMSE value was chosen since it is often considered to indicate excellent performance. Table 6 shows that the proposed model can achieve the targeted performance while requiring 50% fewer parameters than the benchmark model. Table 7 reports the best NMSE performance achieved by the benchmark model as well as the proposed model. The best performance of the proposed CAR-VANN model is obtained with a Tanh–Sigmoid pair of activation functions used in the first and second neural networks of the model, respectively. This results in an NMSE that is slightly superior to that of the benchmark model, with a 35% reduction in the total number of parameters. Similarly, the Sigmoid-ReLU activation functions used in the first and second NN for the proposed model, respectively, lead to the same performance as the benchmark model with only 65% of its complexity. These results clearly showcase the superiority of the proposed model in achieving a much better trade-off between complexity and performance than its single-box ARVTDNN counterpart. It is worth noting here that, as seen in Table 6 and Table 7, the use of the ReLU activation function in the ARVNN part of the CAR-VANN model leads to performances inferior to those of the benchmark model, both in terms of NMSE and complexity. Even though this observation may be specific to the DUT and test conditions, it is essential to ensure that proper activation functions are used in each of the neural networks of the CAR-VANN model since this will impact the overall performance of the model.

**Figure 12:**
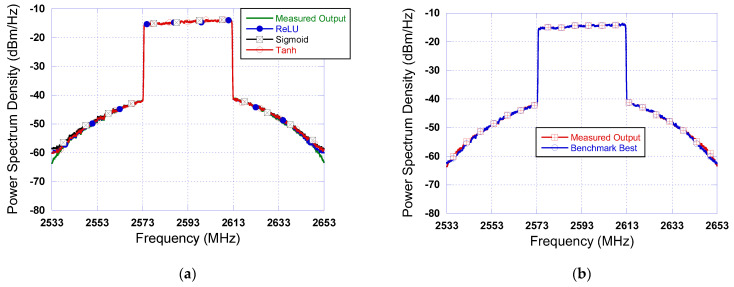

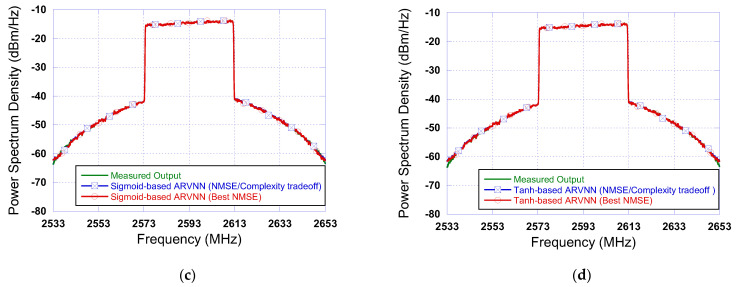
Frequency domain performance benchmarking. (**a**) Benchmark model optimized for NMSE and complexity trade-off. (**b**) Benchmark model optimized for NMSE. (**c**) Proposed model with Sigmoid activation function in the first box. (**d**) Proposed model with Tanh activation function in the first box.

**Figure 13:**
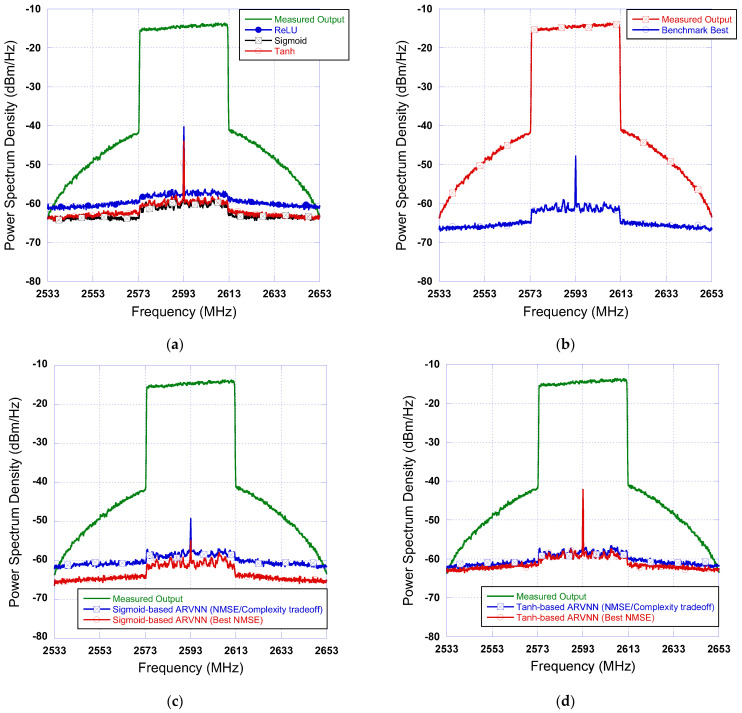
Frequency domain error. (**a**) Benchmark model optimized for NMSE and complexity trade-off. (**b**) Benchmark model optimized for NMSE. (**c**) Proposed model with Sigmoid activation function in the first box. (**d**) Proposed model with Tanh activation function in the first box.

To further assess the proposed model performance and compare it to that of the standalone ARVTDNN in the frequency domain, the spectra measured and estimated at the output of the DUT are reported in Figure 12. First, the spectra predicted using the benchmark model are reported. In Figure 12a, the results shown correspond to the benchmark model optimized for each activation function and for the lowest complexity while ensuring an NMSE that is better than −40 dB. These spectra are obtained for an ARVTDNN with one hidden layer with 30 neurons for the ReLU activation function, two hidden layers with 20 neurons in each for the Sigmoid activation function, and two hidden layers with 15 neurons in each for the Tanh activation function. This corresponds to 752, 922, and 617 trainable parameters for the ARVTDNN using the Relu, Sigmoid, and Tanh activation functions, respectively. Figure 12a shows that the benchmark model accurately predicts the output spectrum across most of the frequency range, with only minor deviations at the band edges. When configured for the lowest NMSE, the benchmark ARVTDNN model leads to an excellent match between the predicted and measured output spectra as depicted in Figure 12b. These results were obtained when a ReLU activation function was used along with two hidden layers with 40 neurons in each, for a total of 2642 parameters. The results of the proposed CAR-VANN model are reported in Figure 12c,d for the cases where the Sigmoid and Tanh activation functions are used in the first box of the CAR-VANN model, respectively. The results are in line with what was observed for the benchmark model. In fact, when the model is optimized for a trade-off between complexity and performance, minor error is observed at the edges of the frequency range. However, the configurations optimized for performance (and achieving an NMSE in the range of −42 dB) demonstrate a consistent match between the model predictions and the measurements. The spectra reported in Figure 12c,d correspond to the models reported in Table 6 for the NMSE/complexity trade-off, and the models reported in Table 7 for the best NMSE curves. Figure 13 presents the frequency domain error corresponding to the data reported in Figure 12. These results are in line with the NMSE results. Accordingly, it is apparent that the proposed model can achieve a performance level comparable to that of the ARVTDNN benchmark at a much lower complexity.

To quantitatively compare the models’ performances reported in Figure 12, the adjacent channel error power ration (ACEPR) was used. The ACEPR quantifies the error in the frequency domain [3], and is defined as

(16) $$ACEPR=10\log_{10}\left(\frac{\int_{f_{os}-\frac{IBW}{2}}^{f_{os}+\frac{IBW}{2}}{\left|Y\left(f\right)-\overset{⌢}{Y}\left(f\right)\right|}^{2}}{\int_{f_{0}-\frac{BW}{2}}^{f_{0}+\frac{BW}{2}}{\left|Y\left(f\right)\right|}^{2}}\right),$$

where $Y\left(f\right)$ and $\overset{⌢}{Y}\left(f\right)$ are the Fourier transform of the measured and estimated signals (y and $\overset{⌢}{y}$), respectively. $f_{0}$ and BW refer to the center frequency and the bandwidth of the input signal, respectively. These are used to compute the channel power. The error power in the adjacent channel is calculated around an offset frequency $f_{os}$ over an integration bandwidth (IBW). The values of $f_{os}$ and IBW can be adjusted to calculate the ACEPR in the lower and upper adjacent or alternate adjacent channels. In this work, ACEPR was computed in the adjacent channel with an offset $f_{os}=40\;\mathrm{MHz}$ for the upper adjacent channel (ACEPR_U) and $f_{os}=-40\;\mathrm{MHz}$ for the lower adjacent channel (ACEPR_L), with IBW=40 MHz in both cases. The ACEPR was not computed in the alternate adjacent channel since the sampling rate used does not offer enough observation bandwidth to capture the full extent of the alternate adjacent channel. The ACEPR results are reported in Table 8 for the spectra of Figure 12. The ACEPR values are consistent with the NMSE performance of the models as reported in Table 4, Table 6 and Table 7.

## 4. Conclusions

In this work, a novel two-box PA model using augmented real-valued neural networks is proposed. The model comprises a memoryless ARVNN to model the memoryless nonlinearity and an ARVTDNN to model the dynamic nonlinearities. The proposed model was experimentally validated and compared to the standalone ARVTDNN model. As is the case for all neural networks, the model performance depends on the appropriate selection of the activation function, among other factors. More specifically, it was observed that the proposed CAR-VANN model does not perform well when the ReLU activation function is used for the ARVNN part of the model, resulting in performances that are inferior to those of the conventional ARVTDNN. However, it was also revealed that when the activation functions are properly selected, the proposed CAR-VANN model achieves an NMSE of −40 dB, with as few as 294 parameters, while a minimum of 617 parameters is needed for the conventional ARVTDNN to obtain an NMSE better than −40 dB. Furthermore, when optimized for the highest accuracy, the proposed model was able to outperform the ARVTDNN while using approximately 35% fewer parameters. Consequently, the proposed work addresses one of the biggest drawbacks faced with neural networks by applying some of the techniques widely utilized for analytically defined PA models.

## Figures and Tables

**Table 1:** Summary of variables definition.

Neural Network	Variable	Definition
First Neural Network (Augmented Real-Valued Neural Network)	$x_{in}$	Input signal
	$K_{1}$	Nonlinearity order for the input features vector
	$L_{ARVNN}$	Number of hidden layers
	$N_{i,ARVNN}$	Number of neurons in the *i*th layer
	$C_{ARVNN}$	Total number of weights and biases
Second Neural Network (Augmented Real-Valued Time-Delay Neural Network)	$x_{\mathrm{int}}$	Input signal
	$K_{2}$	Nonlinearity order for the input features vector
	$L_{ARVTDNN}$	Number of hidden layers
	$M_{0}$	Memory depth associated with $I_{\mathrm{int}}$ and $Q_{\mathrm{int}}$
	$M_{i}$	Memory depth associated with ${\left|x_{\mathrm{int}}\right|}^{i}\;\;\;i=1,2,\ldots ,K_{2}$
	$N_{i,ARVTDNN}$	Number of neurons in the *i*th layer
	$C_{ARVTDNN}$	Total number of weights and biases

**Table 2:** Summary of model training settings.

Parameter	Value
Optimizer	Adam
Learning rate $\left(\eta \right)$	2 × 10^−3^
Training loss function	Mean Squared Error
Dataset split	60% train/20% validation/20% test
Input/output normalization	Maximum Absolute
Batch size	256
Training epochs $\left(E_{\max}\right)$	200
Early stopping criterion	None
Random seed	Not Fixed

**Table 3:** Sweep settings for the training of the neural network.

Parameter	Values/Sweep Ranges
Activation function	ReLU, Sigmoid, Tanh
Number of hidden layers	1, 2, 3, 4
Neurons per hidden layer	5, 10, 15, 20, 25, 30, 35, 40

**Table 4:** Summary of the ARVTDNN model performance.

Activation Function	NMSE	Number of Hidden Layers	Number of Neurons per Hidden Layer	Complexity
ReLU	−40.1 dB	1	30	752
Sigmoid	−40.3 dB	2	20, 20	922
Tanh	−40.7 dB	2	15, 15	617

**Table 5:** Performance summary of the ARVNN model (first neural network of the proposed model).

Activation Function	NMSE	Number of Hidden Layers	Number of Neurons per Layer	Complexity
ReLU	−24.8 dB	1	10	82
Sigmoid	−24.5 dB	1	5	42
Tanh	−24.9 dB	1	5	42

**Table 6:** Performance benchmarking of the CAR-VANN model (trade-off performance vs. complexity).

Parameter	Benchmark	Proposed	Proposed	Proposed
Activation Function #1	Tanh	ReLU	Sigmoid	Tanh
Activation Function #2		ReLU	Sigmoid	Tanh
NMSE	−40.7 dB	−40.5 dB	−40.0 dB	−40.0 dB
Number of Layers	2	1	1	1
Number of Neurons	15	30	10	10
Complexity	617	834	294	294
Relative Complexity	100%	135.2%	47.6%	47.6%

**Table 7:** Performance benchmarking of the CAR-VANN model (best performance).

Parameter	Benchmark	Proposed	Proposed	Proposed
Activation Function #1	ReLU	ReLU	Sigmoid	Tanh
Activation Function #2		ReLU	ReLU	Sigmoid
NMSE	−42.4 dB	−41.5 dB	−42.4 dB	−42.7 dB
Number of Layers	2	2	2	2
Number of Neurons	40	40	30	30
Complexity	2642	2724	1724	1724
Relative Complexity	100%	103.1%	65.3%	65.3%

**Table 8:** Performance summary in time and frequency domains.

Model	Trace	NMSE (dB)	ACEPR_U (dB)	ACEPR_L (dB)
Benchmark	Figure 12a—ReLU	−40.1 dB	−49.6 dB	−49.2 dB
Benchmark	Figure 12a—Sigmoid	−40.3 dB	−50.0 dB	−49.1 dB
Benchmark	Figure 12a—Tanh	−40.7 dB	−50.1 dB	−49.9 dB
Benchmark	Figure 12b—Benchmark Best	−42.4 dB	−54.4 dB	−53.5 dB
Proposed	Figure 12c—NMSE/Complexity Tradeoff	−40.0 dB	−48.7 dB	−48.4 dB
Proposed	Figure 12c—Best NMSE	−42.4 dB	−52.9 dB	−52.4 dB
Proposed	Figure 12d—NMSE/Complexity Tradeoff	−40.0 dB	−49.3 dB	−48.7 dB
Proposed	Figure 12d—Best NMSE	−42.7 dB	−53.5 dB	−53.2 dB

## Data Availability

The data used for this research work are not publicly available.

## References

[1] Ghannouchi F., Hammi O., Helaoui M. (2015). Behavioral Modeling and Predistortion of Wideband Wireless Transmitters.

[2] Morgan D.R., Ma Z., Kim J., Zierdt M.G., Pastalan J. (2006). A Generalized Memory Polynomial Model for Digital Predistortion of RF Power Amplifiers. IEEE Trans. Signal Process..

[3] Barry A., Li W., Becerra J.A., Gilabert P.L. (2021). Comparison of Feature Selection Techniques for Power Amplifier Behavioral Modeling and Digital Predistortion Linearization. Sensors.

[4] Hemsi C.S., Panazio C.M. (2022). Sparse Flexible Reduced-Volterra Model for Power Amplifier Digital Pre-Distortion. IEEE Access.

[5] Crespo-Cadenas C., Madero-Ayora M.J., Becerra J.A., Cruces S. (2022). A Sparse-Bayesian Approach for the Design of Robust Digital Predistorters Under Power-Varying Operation. IEEE Trans. Microw. Theory Tech..

[6] Pedrosa C., Pham D.-K.G., Rashev P., Almairac P., Nanan J.-C., Desgreys P. (2025). Discontinuity Characterization and Low-Complexity Smoothing in RF-PA Polynomial Piecewise Modeling. Sensors.

[7] Langborn B., Fager C., Hou R., Eriksson T. (2026). Concurrent Multi-Beam Digital Predistortion Using FFT Beamforming and Virtual Arrays. Sensors.

[8] Shahghasi A., Montoro G., Gilabert P.L. (2025). Digital Self-Interference Cancellation Strategies for In-Band Full-Duplex: Methods and Comparisons. Sensors.

[9] Liu T., Boumaiza S., Ghannouchi F.M. (2006). Augmented Hammerstein Predistorter for Linearization of Broad-band Wireless Transmitters. IEEE Trans. Microw. Theory Tech..

[10] Gilabert P., Montoro G., Bertran E. On the Wiener Hammerstein Models for Power Amplifiers Predistortion. Proceedings of the IEEE Asia Pacific Microwave Conference.

[11] Hammi O., Ghannouchi F.M. (2009). Twin Nonlinear Two-Box Models for Power Amplifiers and Transmitters Exhibiting Memory Effects with Application to Digital Predistortion. IEEE Microw. Wirel. Compon. Lett..

[12] Wu J., He S., Peng J., Li C., You F. Power Scalable Behavioral Model in Digital Predistrotion for Power Amplifiers. Proceedings of the IEEE Asia Pacific Microwave Conference.

[13] Hammi O., Kwan A., Bensmida S., Morris K.A., Ghannouchi F.M. (2014). A Digital Predistortion System with Extended Correction Bandwidth with Application to LTE-A Nonlinear Power Amplifiers. IEEE Trans. Circuits Syst. I Reg. Pap..

[14] Abdelnaby M., Alnajjar R., Bensmida S., Hammi O. (2024). Reduced Complexity Sequential Digital Predistortion Technique for 5G Applications. Smart Cities.

[15] Spano C., Badini D., Cazzella L., Matteucci M. (2025). Local and Remote Digital Pre-Distortion for 5G Power Amplifiers with Safe Deep Reinforcement Learning. Sensors.

[16] Wu H., Chen W., Liu X., Feng Z., Ghannouchi F.M. (2022). A Uniform Neural Network Digital Predistortion Model of RF Power Amplifiers for Scalable Applications. IEEE Trans. Microw. Theory Tech..

[17] Rawat M., Rawat K., Ghannouchi F.M. (2010). Adaptive Digital Predistortion of Wireless Power Amplifiers/Transmitters using Dynamic Real-Valued Focused Time-Delay Line Neural Networks. IEEE Trans. Microw. Theory Tech..

[18] Rosołowski D.W., Jędrzejewski K. Experimental Evaluation of PA Digital Predistortion Based on Simple Feedforward Neural Network. Proceedings of the 2020 23rd International Microwave and Radar Conference.

[19] Wang D., Aziz M., Helaoui M., Ghannouchi F.M. (2019). Augmented Real-Valued Time-Delay Neural Network for Compensation of Distortions and Impairments in Wireless Transmitters. IEEE Trans. Neural Netw. Learn. Syst..

[20] Hu X., Liu Z., Yu X., Zhao Y., Chen W., Hu B., Du X., Li X., Helaoui M., Wang W. (2022). Convolutional Neural Network for Behavioral Modeling and Predistortion of Wideband Power Amplifiers. IEEE Trans. Neural Netw. Learn. Syst..

[21] Jaraut P., Abdelhafiz A., Chenini H., Hu X., Helaoui M., Rawat M., Chen W., Boulejfen N., Ghannouchi F.M. (2021). Augmented Convolutional Neural Network for Behavioral Modeling and Digital Predistortion of Concurrent Multiband Power Amplifiers. IEEE Trans. Microw. Theory Tech..

[22] Jaraut P., Dhar S., Helaoui M., Boulejfen N., Rawat M., Chen W., Rawat K., Outaleb N., Ghannouchi F.M. (2023). Behavioral Modeling and Digital Predistortion of Mismatched Wireless Transmitters using Convolution Neural Networks. IEEE Trans. Circuits Syst. II Express Briefs.

[23] Ali A., Hammi O. (2023). Bandwidth, Power and Carrier Configuration Resilient Neural Networks Digital Predistorter. IEEE Access.

[24] Sun J., Shi W., Yang Z., Yang J., Gui G. (2019). Behavioral Modeling and Linearization of Wideband RF Power Amplifiers using BiLSTM Networks for 5G Wireless Systems. IEEE Trans. Veh. Technol..

[25] Fischer-Buhner A., Anttila L., Dev Gomony M., Valkama M. (2024). Recursive Neural Network with Phase-Normalization for Modeling and Linearization of RF Power Amplifiers. IEEE Microw. Wirel. Technol. Lett..

[26] Liu M., Yang X., Gao J., Cao S., Liao G., Hou G., Gao D. (2025). Neural Network-Assisted DPD of Wideband PA Nonlinearity for Sub-Nyquist Sampling Systems. Sensors.

[27] Alnajjar R., Hammi O. (2025). A Look-up Table Assisted BiLSTM Neural Network Based Digital Predistorter for Wireless Communication Infrastructure. Sensors.

